# The experience of diagnostic radiography students during the early stages of the COVID‐19 pandemic – a cross‐sectional study

**DOI:** 10.1002/jmrs.544

**Published:** 2021-09-04

**Authors:** Gaynor Lawson Jones, Helen York, Olanrewaju Lawal, Richard Cherrill, Sarah Mercer, Zoe McCarthy

**Affiliations:** ^1^ College Lane University of Hertfordshire Hatfield UK

**Keywords:** COVID‐19, diagnostic radiography, emotion, student, support

## Abstract

**Introduction:**

The rationale for this study was to ascertain the impact of the COVID‐19 pandemic on Undergraduate B.Sc (Hons) Diagnostic Radiography students at the University of Hertfordshire in the UK. This would provide an ideal opportunity for students to reflect on their experience and indicate if they required additional support from the University.

**Methods:**

An online anonymous cross‐sectional survey was deployed to all year groups (*n* = 283) during the first nationwide lockdown in May 2020, eliciting qualitative responses on questions about the effect of the pandemic; emotions experienced; support required and consideration of their choice of a career in the health sector.

**Results:**

Two hundred and one students responded to the survey, with many having experienced the illness or loss of a loved one. Trying to balance family commitments and study was a concern to some students, as well as struggling with the financial burden of job losses or furlough. Many students commented that educational support was a requirement with the move to online teaching and assessment. The main focus of student responses was the emotions that they had experienced, many of which were negative. Anxiety and fear were commonly expressed feelings, along with sadness and feelings of isolation. A small number of students reported feeling grateful, happy and relieved. An overwhelming majority of respondents commented that they were proud to be healthcare students and they were resolute in wanting to continue the course and join the frontline of the NHS workforce.

**Conclusion:**

Some students have been deeply affected by their experiences of the pandemic, and University support mechanisms should be refined to better assist with their ongoing challenges.

## Graphical abstract

United Kingdom‐based students surveyed regarding their emotions during the first phase of the Covid‐19 pandemic. Particular note was made of their intended career in healthcare and if their decisions had altered.

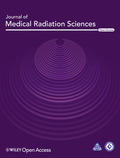

## Introduction

In late December 2019, a number of unexplained serious cases of pneumonia were reported in Wuhan, China. In January 2020, as the number of cases increased, it was confirmed as human‐to‐human transmission caused by a novel coronavirus (2019‐nCoV) infection. The World Health Organisation (WHO) named it Corona Virus Disease 2019 (COVID‐19).^1^

A global pandemic was declared by the WHO on 11 March 2020, and to date, there have been enormous death tolls due to COVID‐19.^2^, ^3^

In March 2020, the government advised the UK population on social distancing measures in order to slow down the spread of the disease in the UK. As this strategy was not reducing COVID‐19 cases, the government advised that the UK would be placed under lockdown from 23 March 2020.^3^, ^4^, ^5^

Public health and government officials introduced social distancing and self‐isolation and asked people to work from home and schools and education centres closed. Health systems introduced emergency measures to cope with the patients exhibiting symptoms of COVID‐19. When the lockdown was implemented, Diagnostic Radiography students were in the midst of clinical placements and final academic weeks prior to the end of year assessments. Placements were suspended and academic study was rapidly converted to online modes of teaching, support, revision sessions and assessments. This occurred across the UK at all institutions providing practical healthcare programmes.

As the pandemic progressed, it became apparent that the student cohort were experiencing increasing difficulties, needed support and reassurance and a new style of guidance for their education. The research team decided to explore the impact of the COVID‐19 pandemic on the student cohort to understand the range of issues experienced and the emotional response to those issues. Support requirements and mechanisms were also addressed in an effort to understand the effects on the education and mental health of students.

There has been a wide variance in infection rates across the country with the London area experiencing an early peak. This is of particular interest as the University is positioned just north of London and a large proportion of the student cohort is based in the wider London area.

This study aims to explore the impact the pandemic has had on the lives and the education of Diagnostic Radiography students by undertaking a qualitative survey of students enrolled on the 3‐year Radiography programme at the University of Hertfordshire, in May 2020. The majority of the literature published on this topic had emerged from China and South East Asia at this point in time, and this study aims to explore the UK student experience.

## Methods

An open‐ended, cross‐sectional qualitative survey was undertaken to gather students’ opinions at a challenging time, with the deployment of an online, anonymous survey to students in all year groups of the Diagnostic Radiography and Imaging programme at the University of Hertfordshire.

All students (*n* = 283) were emailed a link to the survey, which was introduced with a participant information sheet, outlining the rationale behind the study, as well as contact details of the Lead Researcher.^6^ The survey was open for 6 weeks from May 2020. The online survey was used due to the need for timely deployment in a rapidly evolving pandemic. The survey questions (Supporting Information) were designed to elicit free‐text answers and therefore generate qualitative responses about student experiences and emotions during the COVID‐19 pandemic period. Prior to deployment, all questions were reviewed and refined in consultation with the programme Research Lead to ensure the approach was appropriate.^7^

Demographic information was requested with more in‐depth responses required for questions about personal experiences and emotions felt. Respondents were also asked about the support they would have liked and if the pandemic had changed their decision to study towards a career in healthcare.

Ethical approval for the study was gained through the Health, Science, Engineering and Technology Ethics Committee with Delegated Authority (ECDA). Consideration and acknowledgement were given to the potential that students may have had challenging situations or even bereavement as part of their experiences, so the details of the University Student Wellbeing department were provided. Students were also made aware of the availability of staff support, for example, their personal tutor or year tutor. Email reminders were sent over a 4‐week period to ensure that the response rate was optimised.^8^

Qualitative data were analysed using inductive clustering to derive themes from the data, and rigour was assured by having two different researchers coding responses and extracting themes independently for each question. Different pairs of researchers worked on each question to minimise bias and increase validity.^9^ The research team met regularly to triangulate findings and compare emergent themes, something deemed important, given the potentially sensitive nature of the information sought. This allowed for any additional support measures to be provided to students in a timely manner.

## Results

Not all students responded to all questions.

### Demographics

**Figure 1:**
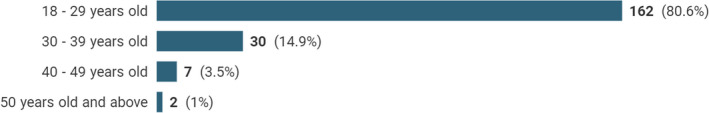
Respondent age.

Two hundred and eighty‐three undergraduate Diagnostic Radiography students were sent an anonymous online survey link and 71% responded (*n* = 201). Nearly 50% of respondents were year 1 students (*n* = 99), with 37% being second‐year students (*n* = 75), and the remainder being third‐year students (*n* = 27). There is a predominance of females in the respondents (66.7%), 31.3% identifying as male, and 2% preferring not to state their gender (Figure 1).

The majority of respondents are aged between 18 and 29 years old (80%) with 15% aged 30–39; 3.5% aged between 40 and 49, and there were two respondents over 50.

When asked where they were resident during the COVID‐19 period, 121 students responded with ‘home’ which gave little in terms of geographical demographics. Hatfield and London were identified by 50 students, and 11 students mentioned that they were in placement accommodation before travelling home. Other students responded that they were within a 50‐mile radius of Hatfield, with 3 in ‘the North’ and 3 overseas.

A considerable number of respondents (*n* = 40) indicated that they had caring responsibilities and the majority of these stated that they had children.

### Impact of COVID‐19

A large number of respondents indicated that they, or their family, had been directly impacted by COVID‐19. Students who answered positively to this question were then asked to elaborate on their feelings in relation to this.

Participants indicated that they or family members had been unwell with COVID‐19, with some indicating that family members had been hospitalised, and a number of respondents noted that family members or friends had died from the virus.

My grandad passed away from Covid. It was awful and still is

I lost my younger sister with COVID‐19

deaths‐prefer not to comment regarding feelings thank you

Financial concerns or job losses were also reported by respondents.

…we have been severely affected financially.

…we are now struggling with money. I might need to leave university.

Respondents indicated that they were impacted by having to home school children or that family members were frontline staff in the NHS.

my children have been spending their time at home (24/7) which has directly affected my ability to participate in all aspects of the course to the level expected.

Brother works for the NHS. Saw people die in front of him… sleeping at work

Isolation and a deterioration in mental health was described by a number of participants and the impact this had on their education and placement was also mentioned.

Being isolated was emotionally distressing,… affected financially.

Being kept in isolation …severely impacted my mental health.

Falling ill to COVID‐19 during exams

### Perceived support requirements

Qualitative responses from respondents indicated their needs were based on educational, counselling and pastoral support, while some respondents indicated the need for financial and childcare support, with a small number mentioning food parcels. Timely communication of any changes made to the course delivery was also highlighted.

Conversely a small number of respondents reported being happy with everything that had been provided and did not feel the need for anything further.

I don't think any amount of support could have helped me find peace at that time. I just needed some time to myself to reflect.

bereavement counselling

The university was very supportive I didn’t need any other help

### Emotions

When asked to describe the types of emotions experienced from mid‐March to mid‐May 2020, a large majority of respondents stated anxiety and fear as something they were feeling.

In addition, many reported sadness as one of the emotions they had experienced.

Anxiety, Fear for my family and myself, Isolation, Heightened emotions

…whirlwind of emotions I've experienced these past few months, nothing short of anxiety, depression, loss of appetite and insomnia.

…great sadness and pain.

A common set of emotions noted by respondents included boredom, tiredness or loneliness.

…I feel totally worn out and totally exhausted.

…I’ve lost track of days, everyday feels the same.

Feelings of loneliness and isolation.

A number of participants expressed anger and frustration as emotions they had felt in the previous 3 months.

For the last three months, I have felt very low – anger, sadness…

Not all emotions reported by respondents were negative. Some commented that they were happy and optimistic, with some further commenting that they felt focussed and purposeful.

Feelings of contentment Feeling focused Feeling grateful

Happiness, enjoyment, relief

### Choice of career

Participants were asked whether this experience and their associated feelings had affected their thoughts of their career choice. The qualitative responses (*n* = 117) were then analysed and subcategorised into positive and negative themes. The majority of participants stated that they want to continue their training to be able to help people, with a large number stating they were passionate and proud of their chosen career.

A very small number stated that they had considered withdrawing from the course.

If anything, this has made me want to become a health professional even more

I am still proud to be a healthcare professional. It’s a privilege to care for people

## Discussion

This study elicited detailed responses of students’ experience during the early part of the COVID‐19 pandemic. The response rate of 71% (*n* = 201) of participants was acknowledged by the researchers as an exceptional achievement and it was felt this indicated the depth of feeling from the student cohort. It was recognised that the timing of the survey 8 weeks into the United Kingdom lockdown probably impacted this response rate as students appreciated an opportunity to voice their concerns and feelings.

The review of the literature on the effect of COVID‐19 on radiography students shows that a small number of studies have examined this specific group.^10^, ^11^ Most of the studies available were with nursing and medical students, so some comparisons can be drawn related to the radiography student population. All these studies used a closed‐ended questionnaire to examine the topic, which limits their data.^12^, ^13^ The main benefit of this study is that it provides some explanation for the issues identified in the previous studies. The results of this study are categorised into three overall themes – information, emotion and support. These categories are explored together with the findings of the research below.

### Emotions

This theme consists of both negative and positive feelings. It was noted that the participants' emotions are linked to their confidence in the information provided by the government and their learning institution.

### Emotions: negative

When asked about the impact of COVID‐19, the overwhelming responses were about family members and friends being affected by the virus, experiencing illness and hospitalisation and suffering bereavements. Students clearly indicated that the stress and emotion surrounding the infection were impacting their families, friends and themselves. This is supported by studies from China which reported that their participants experienced detrimental effects whilst living in lockdown. They also noted, however, that lockdown might buffer against the anxiety as participants perceive it as a safe approach to a pandemic.^12^, ^14^, ^15^, ^16^, ^17^

One study reported that levels of anxiety were highest in the 21‐ to 40‐year age group^18^ and this correlates with the demographic of the students in this study who are predominantly in this age group.

Studies have shown that anxiety is directly related to the participants’ closeness to the pandemic in terms of geography and the effect on people known to them. It was also noted that participants from cities showed more anxiety and fear than participants from rural areas.^12^, ^14^, ^15^ At the start of the pandemic in the UK, London and its surrounding areas experienced a rapid rise in cases. The University is located just north of London and many of the students live in and around this area and this would have impacted on the high levels of anxiety.

The effect of grief when a normal grief cycle is disrupted is mentioned in literature.^19^, ^20^ The students declared their overwhelming sadness at being unable to grieve and have access to a normal family support network.

I lost my sister and nephew. I am really struggling with it.

We lost 2 family members. It was an awful time…

Social interaction was reduced …we could not seek comfort from others.

A number of studies also highlighted the underlying emotions of anger, depression and sadness as part of professional life. Health professionals need to be prepared to endure these emotions as well as cope with unresolved grief and sadness related to deaths and illness, not only in their workplace but also in their personal life.^18^, ^19^, ^20^

Financial concerns were raised by some students where job losses and furlough schemes were cited either impacting themselves or family members. Several students expressed concern about their ability to provide for their families or meet the financial needs of a university programme.^15^

Some respondents expressed issues of anxiety and concern due to home schooling their children and having family members who were frontline staff. This, in turn, exacerbated their emotional state and stress levels when trying to cope with the switch to online learning and their impending online assessments. They expressed feelings of being torn by their responsibilities of being parents, healthcare students and trying to support the educational needs of their children. During the 2003 Severe Acute Respiratory Syndrome (SARS) outbreak, many nurses experienced the conflict of being a healthcare provider whilst also being a parent. Their concerns related to the need to be a healthcare professional and the anxieties of protecting their families from infection.^12^, ^17^

Studies have explored student’s experiences during the early stages of COVID‐19 and found that isolation and being separated from close family networks had a significant impact on their mental health and anxiety.^15^, ^21^, ^22^ This closely reflected the responses received in this study. Students stated that they struggled with feelings of isolation and loneliness and described significant changes in levels of sadness, depression and feelings of separation. Other emotions stated were anger, fear, being unable to concentrate or feeling overwhelmed.

### Emotions: positive

A surprising number of students indicated that, although they had experienced mixed emotions, they had experienced happiness, along with a sense of gratitude and relief. Feeling purposeful and optimistic were also reported. Positive emotions have rarely been reported, as it seems that measuring the intensity of negative emotions has been more of a focus in literature, rather than gathering the entire range of emotions experienced.^14^, ^15^, ^16^, ^18^, ^19^, ^20^

The majority of respondents revealed that they were happy with their choice of career. They commented that this is their chosen career and they enjoy what they do and want to continue to help people. The public appreciation of healthcare professionals during the pandemic may have validated their confidence. Several students specifically stated that when registering for a health professions course, they knew that there was always the possibility of a pandemic occurring and they were prepared for this. A prominent feature of the qualitative responses was that students were feeling a definite sense of pride in their role as student radiographers and that they have found the situation very rewarding. Other studies relating to students in healthcare have reported similar findings in the attitudes of their students.^17^, ^23^

### Support

When asked about the support that they would have liked, a large number of students stated that they needed support with their educational needs, with less stating counselling or pastoral support was required. Specific comments were made relating to how exams would be adjusted to an online format and wanting more online revision sessions to reflect the changes in assessment. Literature shows that students value support from institutions, family and friends and these were essential sources of support and comfort whilst students struggled to cope with the stress and anxiety associated with studying online.^14^, ^15^, ^17^, ^23^, ^24^ The expectation is that institutions should provide psychological support to students or refer them to the appropriate organisations that could help them overcome their problems.^23^, ^24^, ^25^ Interestingly, some students stated that they would have appreciated a higher level of reassurance from the University; however, given the dynamic nature of the pandemic, this would have been difficult to achieve. A few students also requested basic guidance on how to cope emotionally while being isolated from family and friends. Respondents also mentioned that misinformation had caused additional stress. Training in coping strategies and regular exercises might help students improve their ability to control their emotions and feelings.^12^, ^16^, ^26^

Students stated that they would have liked greater guidance from the University about their academic and placement changes. This was challenging due to the daily changing landscape, but it is an issue to be considered in the future. The need for targeted support for students undertaking placement has been noted by other studies, indicating a consistency in the students’ perception of support requirements.^23^, ^26^

It was gratifying to note that several students considered the level and type of support given was adequate. There were also respondents who were unsure of their needs as the situation was so changeable.

### Information

A key factor in this study is information, as this could influence the students’ emotions and access to support. At the start of the pandemic, information was changing rapidly and circulated through multiple sources. The need for accurate and supportive information was reported in a number of studies that mention the impact of social media, and the anxiety it can evoke in students. In addition, the effects of distressing news reports, rumours and fabricated reporting of COVID‐19 resulted in anxiety and fear and the participants of this study reported similar feelings related to information sources.^14^, ^18^, ^24^, ^27^, ^28^

Restrictions on face‐to‐face teaching resulted in most teaching and assessment being delivered online. The use of online delivery has its challenges, as individuals who are less technically literate may struggle, and students who do not have ready access to internet or laptops might not be able to participate consistently,^24^, ^29^ Respondents reported concerns about using new technology and coping with education online. When the students were advised of the lockdown process, the information and University advice for them were changing on an almost daily basis. The need to move to online education and the impact this has on the anxiety levels of students was explored in a number of studies and students predominantly expressed anxiety about the future unfamiliarity and uncertainty.^24^, ^30^, ^31^, ^32^, ^33^, ^34^, ^35^, ^36^, ^37^, ^38^, ^39^ ^9^ A review of how information was provided to students would be useful to establish the best platforms to share constant, and ongoing updates.

### Recommendations and questions

Consideration needs to be given to whether students are being adequately prepared for working in the NHS, in light of the overwhelming impact of COVID‐19 on healthcare provision. Universities will need to develop curricula to integrate specific content related to pandemics and infection control. Consideration must also be given to how placements can be adjusted so that students can maintain experience in an effective and safe way. Preparing students to engage successfully with an online learning environment should also be prioritised, as societal restrictions may be in place long term.

Pastoral and financial support for students should be closely reviewed to ensure it is adequate and provided in a timely manner. Successful management strategies and approaches to student well‐being, particularly in healthcare students, are imperative in ensuring the students complete their programme of study within a normal timeframe. The NHS is reliant on new practitioners entering their respective professions and any barriers to this should be overcome as a priority.

## Conclusion

COVID‐19 has impacted on everyday life worldwide. This study set out to explore the impact the pandemic has had on the lives and the education of Diagnostic Radiography students and this has been shown to be far reaching and diverse. The participants in this study experienced similar emotions and difficulties to other healthcare students in previous epidemics, such as SARS. Due to the limited research on the COVID‐19 pandemic at the time of this survey, it is difficult to establish whether the findings are unique to radiography students within a pandemic situation. By far the most substantial impact of COVID‐19 has been the emotional impact caused by illness or death within families and friends and the stresses of dealing with these. An ongoing concern is the impact on the mental health and well‐being of these students as they continue their studies and on to employment.

## Conflict of Interest

The authors declare no conflict of interest.

## Supporting information


**Appendix S1**. Survey questions.

## References

[1] World Health Organisation Mission summary: WHO Field Visit to Wuhan, China 20–21 January 2020. Available from: https://www.who.int/china/news/detail/22‐01‐2020‐field‐visit‐wuhan‐china‐jan‐2020. Accessed 21 October 2020.

[2] Worldometer Countries where COVID‐19 has spread, 2020. Available from: https://www.worldometers.info/coronavirus/countries‐where‐coronavirus‐has‐spread/. Accessed 21 October 2020.

[3] Gov.UK Coronavirus (COVID‐19) in the UK UK Summary, 2020. Available from: https://www.Coronavirus.data.Gov.uk. Accessed 19 October 2020.

[4] Nuffield Trust Chart of the Week: The Steep Rise and Slow Decline of COVID‐19 Cases Across the UK, 2020 [cited 2020 November]. Available from: https://www.nuffieldtrust.org.uk/. Accessed 19 October 2020.

[5] GOV.UK Prime Minister's Statement on Coronavirus (COVID‐19): 23 March 2020, 2020 [cited 2020 November]. Available from: https://www.gov.uk/government/speeches/pm‐address‐to‐the‐nation‐on‐coronavirus‐23‐march‐2020. Accessed 18 November 2020.

[6] JISC . Powering world‐class education and research, 2020 [cited 2020 November]. Available from: https://www.jisc.ac.uk/. Accessed 17 July 2020.

[7] Creswell JW , Poth CN . Qualitative Inquiry & Research Design: Choosing Among Five Approaches (International student), 4th edn. Sage, Los Angeles, CA, 2018.

[8] Beatty PC , Collins D , Kaye L , Willis GB , Wilmot A , Padilla JL . Advances in Questionnaire Design, Development, Evaluation and Testing. John Wiley & Sons, Incorporated, Newark, NJ, 2019.

[9] Silverman D . Interpreting Qualitative Data, 5th edn. Sage, London, 2014.

[10] Teo LW , Pang T , Ong YJ , Lai C . Coping with COVID‐19: perspectives of student radiographers. J Med Imaging Radiat Sci 2020; 51: 358–60. 32571652 10.1016/j.jmir.2020.05.004 PMC7256620

[11] Akudjedu TN , Lawal O , Sharma M , et al. Impact of the COVID‐19 pandemic on radiography practice: findings from a UK radiography workforce survey. BJR Open 2020; 2: 20200023. 33178980 10.1259/bjro.20200023 PMC7583354

[12] Huang L , Lei W , Xu F , Liu H , Yu L . Emotional responses and coping strategies in nurses and nursing students during COVID‐19 outbreak: a comparative study. PLoS One 2020; 15: e0237303. 32764825 10.1371/journal.pone.0237303 PMC7413410

[13] Maltby J . Research Methods for Nursing and Healthcare. Pearson Education, Harlow, 2010.

[14] Li Y , Wang Y , Jiang J , et al. Psychological distress among health professional students during the COVID‐19 outbreak. Psychol Med 2020: 11: 1–3. 10.1017/S0033291720001555. PMC7225209 32389148

[15] Cao W , Fang Z , Hou G , et al. The psychological impact of the COVID‐19 epidemic on college students in China. Psychiatry Res 2020; 287: 112934. 32229390 10.1016/j.psychres.2020.112934 PMC7102633

[16] Zhang Y , Zhang H , Ma X , Di Q . Mental health problems during the COVID‐19 pandemics and the mitigation effects of exercise: a longitudinal study of college students in china. Int J Environ Res Public Health 2020; 17: 3722. 10.3390/ijerph17103722 PMC7277113 32466163

[17] Gallagher TH , Schleyer AM . “We signed up for this!” – student and trainee responses to the COVID‐19 pandemic. N Engl J Med 2020; 382: e96. 32268020 10.1056/NEJMp2005234

[18] Salari N , Hosseinian‐Far A , Jalali R , et al. Prevalence of stress, anxiety, depression among the general population during the COVID‐19 pandemic: a systematic review and meta‐analysis. Global Health 2020; 16: 57. 32631403 10.1186/s12992-020-00589-w PMC7338126

[19] Mortazavi SS , Assari S , Alimohamadi A , Rafiee M , Shati M . Fear, loss, social isolation, and incomplete grief due to COVID‐19: a recipe for a psychiatric pandemic. Basic Clin Neurosci 2020; 11: 225–32. 32855782 10.32598/bcn.11.covid19.2549.1 PMC7368098

[20] Assari S , Habibzadeh P . The COVID‐19 emergency response should include a mental health component. Arch Iran Med 2020; 23: 281–2. 32271604 10.34172/aim.2020.12

[21] Elmer T , Mepham K , Stadtfeld C . Students under lockdown: comparisons of students’ social networks and mental health before and during the COVID‐19 crisis in Switzerland. PLoS One 2020; 15: e0236337. 32702065 10.1371/journal.pone.0236337 PMC7377438

[22] Rajkumar RP . COVID‐19 and mental health: a review of the existing literature. Asian J Psychiatr 2020; 52: 102066. 32302935 10.1016/j.ajp.2020.102066 PMC7151415

[23] Collado‐Boira EJ , Ruiz‐Palomino E , Salas‐Media P , Folch‐Ayora A , Muriach M , Baliño P . “The COVID‐19 outbreak” – an empirical phenomenological study on perceptions and psychosocial considerations surrounding the immediate incorporation of final‐year Spanish nursing and medical students into the health system. Nurse Educ Today 2020; 92: 104504. 32563039 10.1016/j.nedt.2020.104504 PMC7289744

[24] Sahu P . Closure of universities due to coronavirus disease 2019 (COVID‐19): impact on education and mental health of students and academic staff. Curēus 2020; 12: e7541. 32377489 10.7759/cureus.7541 PMC7198094

[25] Pragholapati A . COVID‐19 Impact on Students. Open Science Framework, Bandung, Indonesia: Universitas Pendidikan Indonesia, 2020. 10.17605/osf.io/nuyj9.

[26] Glauser W . Uncalm before the storm. Can Med Assoc J 2020; 192: E417–8. 32392508 10.1503/cmaj.1095860 PMC7162431

[27] Ferrel MN , Ryan JJ . The impact of COVID‐19 on medical education. Curēus 2020; 12: e7492. 32368424 10.7759/cureus.7492 PMC7193226

[28] De Kock JH , Latham HA , Leslie SJ , et al. A rapid review of the impact of COVID‐19 on the mental health of healthcare workers: implications for supporting psychological well‐being. BMC Public Health 2021; 21: 104. 33422039 10.1186/s12889-020-10070-3 PMC7794640

[29] Lincango‐Naranjo E , Solis‐Pazmino P , Rodriguez‐Villafuerte S , et al. Paradigms about the COVID‐19 pandemic: what medical students know and feel. SSRN Electron J 2020. 10.2139/ssrn.3594642. PMC7903404 33627116

[30] Gao J , Zheng P , Jia Y , et al. Mental health problems and social media exposure during COVID‐19 outbreak. PLoS One 2020; 15: e0231924. 32298385 10.1371/journal.pone.0231924 PMC7162477

[31] Baloran ET . Knowledge, attitudes, anxiety, and coping strategies of students during COVID‐19 pandemic. J Loss Trauma 2020; 25: 635–42.

[32] Toquero CM . Challenges and opportunities for higher education amid the COVID‐19 pandemic: the Philippine context. Pedagog Res 2020; 5: em0063.

[33] Rose S . Medical student education in the time of COVID‐19. JAMA J Am Med Assoc 2020; 323: 2131–2. 10.1001/jama.2020.5227 32232420

[34] Raj U , Fatima A . Stress in students after lockdown due to COVID‐19 threat and the effects of attending online classes. SSRN Electron J 2020. 10.2139/ssrn.3584220.

[35] Wanigasooriya K , Palimar P , Naumann DN , et al. Mental health symptoms in a cohort of hospital healthcare workers following the first peak of the COVID‐19 pandemic in the UK. BJPsych Open 2021; 7: e24. 10.1192/bjo.2020.150 PMC7844153 33371927

[36] Boelen PA , Spuij M , Lenferink LIM . Comparison of DSM‐5 criteria for persistent complex bereavement disorder and ICD‐11 criteria for prolonged grief disorder in help‐seeking bereaved children. J Affect Disord 2019; 250: 71–8. 30836282 10.1016/j.jad.2019.02.046

[37] Gallo LA , Gallo TF , Young SL , Moritz KM , Akison LK . The impact of isolation measures due to COVID‐19 on energy intake and physical activity levels in Australian university students. Nutrients 2020; 12: 1865. 10.3390/nu12061865 PMC7353248 32585830

[38] Lasalvia A , Bonetto C , Porru S , et al. Psychological impact of COVID‐19 pandemic on healthcare workers in a highly burdened area of north‐east Italy. Epidemiol Psychiatr Sci 2021; 30: e1. 10.1017/S2045796020001158 PMC7804082 33331255

[39] Wald HS , Monteverde S . COVID‐19 era healthcare ethics education: cultivating educational and moral resilience. Nurs Ethics 2021; 28: 58–65. 33427018 10.1177/0969733020976188

