# Supplementary material for: The experience of diagnostic radiography students during the early stages of the COVID‐19 pandemic – a cross‐sectional study
Source: J Med Radiat Sci. 2021 Sep 4;68(4):418–25. doi: 10.1002/jmrs.544 (PMC8655757; doi:10.1002/jmrs.544)
Supplement: Supplementary file 1 — Appendix S1. Survey questions. [file JMRS-68-418-s001.docx]

**Supporting Information**

Title of study: Experiences of diagnostic radiography students during the COVID-19 period

**Section A: Demographics**

1. What is your current year of study?
2. Year 1
3. Year 2
4. Year 3
5. What is your age?

a. 18 - 29 years old

b. 30 - 39 years old

c. 40 - 49 years old

d. 50 years old and above

1. What is your gender?

a. Male

b. Female

c. Prefer not to say

4. Where were you residing during the COVID-19 period?

5. Do you have any current caring responsibilities/dependents?

a. Yes

b. No

6. If yes to the above, please provide additional comments regarding caring responsibilities in the box below.

**Section B: Impact of COVID-19**

Have you or your family been directly impacted by COVID-19? Please think about how you and/or your family were affected and tell us about your experience.

For each experience, tell us how that made you feel at the time and now.

Tell us what support you would have liked to have at that time and now.

Please can you tell us the types of emotions you have experienced in the last 3 months

Do you feel you would have felt differently had you not been on a healthcare programme?

Reflection - Thoughts and feelings

Reflecting on these emotions, what are your thoughts now about choosing a career as a health professional?

Has this experience and your feelings affected your choice of career at all? Tell us why…

Thank you very much for your time.
